# Unraveling Heterogeneity of Tumor Cells and Microenvironment and Its Clinical Implications for Triple Negative Breast Cancer

**DOI:** 10.3389/fonc.2021.557477

**Published:** 2021-03-29

**Authors:** Ke Jiang, Mengting Dong, Chunyang Li, Jiayu Sheng

**Affiliations:** Department of Breast Diseases, Yueyang Hospital of Integrated Traditional Chinese and Western Medicine, Shanghai University of Traditional Chinese Medicine, Shanghai, China

**Keywords:** triple negative breast cancer, single-cell RNA-seq, immune cells, heterogeneity, tumor microenvironment

## Abstract

**Objective:** Triple negative breast cancer (TNBC) is an aggressive subtype of breast cancer, characterized by extensive intratumoral heterogeneity. We aimed to systematically characterize the tumor heterogeneity of TNBC.

**Methods:** Single-cell RNA sequencing (scRNA-seq) of TNBC cells were obtained from the GSE118389 and GSE75688 datasets. After integration of the two datasets, cell clustering analysis was performed using the Seurat package. According to the marker genes of cell cycle, cell cycle of each cell cluster was determined. Then, function enrichment analysis of marker genes in each cell cluster was performed, followed by ligand–receptor signaling network analysis. CIBERSORT was used to estimate the proportion of 22 immune cells in each sample based on RNA-seq data of 58 normal adjacent tissues and 101 TNBC tissues. After that, prognostic value of immune cells was assessed.

**Results:** In the integrated datasets, five cells types including B cells, myeloid cells, stromal cells, T cells, and tumor cells were clustered. Functional enrichment analysis revealed the functional heterogeneity of genes in each cell. Intercellular communication networks were conducted based on ligand–receptor pairs. The heterogeneity in the fractions of 22 immune cells was found in TNBC tissues. Furthermore, there was a significant difference in the fractions of these immune cells between adjacent normal tissues and TNBC tissues. Among them, M2 macrophages and neutrophils were significantly associated with clinical outcomes of TNBC. Moreover, the fractions of T cells CD4 memory resting, monocytes, neutrophils, M1 macrophages, and T cells CD4 memory activated were significantly correlated with clinical characteristics of TNBC. As shown in PCA results, these immune cells could significantly distinguish TNBC tissues into adjacent normal tissues.

**Conclusion:** Our findings characterized the tumor heterogeneity of TNBC, which deepened the understanding of the complex interactions between tumor cells and their microenvironment, especially immune cells.

## Introduction

TNBC is an aggressive subtype of breast cancer (accounting for 12–18%) (1). TNBC patients are often more likely to develop local recurrence and distant metastases than other types (2). As expected, patients with TNBC have worse clinical outcomes. Due to lack of estrogen receptor (ER), progesterone receptor (PR) and HER2 receptors, patients with TNBC are not sensitive to hormone or anti-Her2 therapy. But TNBC is more sensitive to chemotherapy, thus, chemotherapy has become a critical treatment. Yet, as a heterogeneous disease, biomarkers are still lacking for individualized treatment of TNBC, especially immunotherapy (3). Given the high heterogeneity of TNBC, an understanding of the molecular pathogenesis is crucial for developing novel therapeutic strategies.

Single-cell RNA-seq has confirmed the extensive intratumoral heterogeneity in TNBC, which may become a promising clinical application for TNBC therapy. As previous studies, through single-cell RNA-seq, genes resistant to neoadjuvant chemotherapy have been detected for TNBC (4). With the development of single-cell RNA-seq, tumor cells can be monitored to assess tumor heterogeneity and sensitively detect early recurrent tumors as well as rare cell populations. Accurate characterization of tumor transcriptome heterogeneity and gene expression profile of tumor and microenvironment may assist determine sensitive prognostic markers and therapeutic targets (5). Research on tumor heterogeneity may promote the development of molecular targeted therapy of TNBC. The tumor microenvironment composed of cancer-related fibroblasts and immune cells plays a key role in the occurrence, development, and treatment resistance of TNBC (2, 6, 7). The tumor microenvironment has a profound impact on the immunotherapy of TNBC. Thus, the characterization of tumor-infiltrating immune cells may provide better strategies to overcome immunosuppression.

Collectively, in this study, we analyzed the intratumoral heterogeneity and cell-to-cell communication in TNBC. Furthermore, immune cell compositions and their clinical implications were explored. Our study provided a deeper understanding of the heterogeneity of TNBC.

## Materials and Methods

### Acquisition of Single-Cell RNA-seq Data

Single-cell RNA-seq data of 205 TNBC cells from five TNBC patients were obtained from the Gene Expression Omnibus database (GEO; accession: GSE75688) (5). Furthermore, single cell RNA-seq of 1,534 cells in six fresh TNBC tumors from the GSE118389 dataset was also downloaded.

### Quality Control

Because the GSE118389 dataset had been quality controlled, quality control was only presented on the GSE75688 dataset. The DropletUtils package in R was used to detect the gene expression of each cell, and no barcoded expression was filtered out (8). According to the number of unique molecular identifiers (UMI) of each cell, cells were filtered out again. Using the calculatQCMetrics in the scater package, the gene expression in each cell was counted (9). Then, cells were filtered out with the threshold of the ratio of mitochondrial genes ≤5% and ribosomal genes ≥10%. The NormalizeData in the Seurat package was used to normalize the expression matrix of each cell after filtering (10).

### Principal Component Analysis (PCA)

The first 2,000 genes with the most significant differences among cells were identified through the FindVariableFeatures in the Seurat package. Focusing on these genes in downstream analysis helps highlight biological signals in single-cell RNA-seq. The ScaleData in the Seurat package was then used to linearly scale the single-cell RNA-seq data, which regressed the heterogeneity associated with cell cycle stages or mitochondrial contamination [pbmc < - ScaleData(pbmc, vars.to.regress = “percent.mt”)]. Next, linear dimensionality reduction analysis was presented by the RunPCA in the Seurat package.

### Cell Clustering and Identification of Marker Genes

First, the principal components (PCs) with larger standard deviations (cumulative standard deviation >70%) were selected out. The Seurat package was used to standardize the expression matrix of the GSE118389 dataset after filtration and conversion. The two datasets were merged to obtain a total of 1,687 cells. The integration method of the two datasets was as follows: The TPM expression matrices of the two datasets were first loaded separately. After taking the intersection of the genes of the two datasets, “SeuratObject” was constructed, respectively. The GSE75688 dataset was defined as “reference” and the GSE118389 dataset was defined as “query”. “NormalizeData”, “FindVariableFeatures,” and “ScaleData” were separately performed on the data of the two datasets. The “anchor” of the GSE75688 reference dataset was determined through “FindTransferAnchors.” Then, based on the “anchors” of the reference dataset, the cell type of the “query” dataset was scored and predicted, and the type of each cell of the “query” dataset was defined. The “anchors” of the two datasets was determined through FindIntegrationAnchors. Then based on the “anchors,” the two datasets were merged. The FindNeighbors and FindClusters in the Seurat package were used for cell cluster analysis. *Via* the runUMAP in the Seurat package, dimensionality reduction (Uniform Manifold Approximation and Projection, UMAP) and non-linear dimensionality reduction (t-distributed stochastic neighbor embedding, tSNE) analyses were presented. Through the FindAllMarkers in the Seurat package, marker genes for each cell cluster according to the following screening criteria: |log fold change (FC)| ≥1 and adjusted *p*-value (according to the default false discovery rate adjustment) ≤ 0.05.

### Cell Cycle Analysis

According to the marker genes of cell cycle, the cell cycle of each cell cluster was counted using the Seurat package (11). Then, all cells were divided into G1, G2M, and S phases based on their scores.

### Function Enrichment Analysis and Ligand–Receptor Signaling Network Analysis

Based on the Gene Ontology (GO) database and KEGG pathway database, functional enrichment analysis of marker genes (log2FC ≥ 1, adjusted *p* ≤ 0.05) of each cell type was performed to explore their potential biological functions and pathways (12, 13). GO terms contained biological process (GO-BP), cellular component (GO-CC) and molecular function (GO-MF). *p* < 0.05 was considered significantly enriched. The ligand–receptor pairs in markers of each type of cells were evaluated based on published literature (14). Based on the ligand receptor network diagram of the cell type, the detailed relationship network diagram of the ligand receptor in each cell type and the relationship network diagram of the designated ligand receptor and each cell group were separately constructed. Moreover, the number of ligand–receptor relationship pairs was counted.

### RNA-seq Data

Level 3 RNA-seq data of 1,097 breast invasive carcinoma (BRCA) samples were downloaded from The Cancer Genome Atlas (TCGA; https://cancergenome.nih.gov/) database. TNBC samples were screened as follows: (1) ER (–), PR (–), and HER2 (–); (2) complete clinical information. Finally, a total of 113 adjacent normal samples and 112 TNBC samples were obtained for this study. Clinical information of patients is shown in Table 1. The differences in expression of key genes were analyzed between TNBC and normal tissues *via* Wilcoxon rank-sum test. *p* < 0.05 was considered statistically significant.

**Table 1:** Clinical characteristics of patients with TNBC in the TCGA datasets.

**Clinical characteristics**	**Groups**	**Patient *n* (%)**
Survival status	Alive	100(89.3)
	Dead	12(10.7)
Age	≤65	94(83.9)
	> 65	18(16.1)
Pathologic stage	Stage I	19(16.9)
	Stage II	72(64.3)
	Stage III	19(16.9)
	Stage IV	2(1.8)
Pathologic T	T1	26(23.2)
	T2	71(63.4)
	T3	12(10.7)
	T4	3(2.7)
Pathologic M	M0	96(85.7)
	M1	2(1.8)
	Mx	14(12.5)
Pathologic N	N0	73(65.2)
	N1	25(22.3)
	N2	10(8.9)
	N3	4(3.6)

### Estimation of Immune Infiltration

CIBERSORT (http://cibersort.stanford.edu/), a deconvolution algorithm developed by Newman et al., was used to estimate the abundance of cell types in mixed cell populations based on TNBC RNA-seq data (8). The proportion of 22 immune cells in each sample was calculated and an algorithm was run on 1,000 permutations with the LM22 feature matrix. For each sample, the sum of the proportion of all immune cell types was equal to 1.

### Prognostic Value of Immune Infiltration

To assess the prognostic value of immune cells, cox regression analysis was used to evaluate the correlation between immune cell proportion and survival time. In addition, we investigated the correlation between immune cell proportion and clinical characteristics including stage and pathologic TNM by Spearman test. *p* < 0.05 was considered statistically significant.

## Results

### Quality Control and Preprocessing of Single-Cell RNA-seq

**Figure 1:**
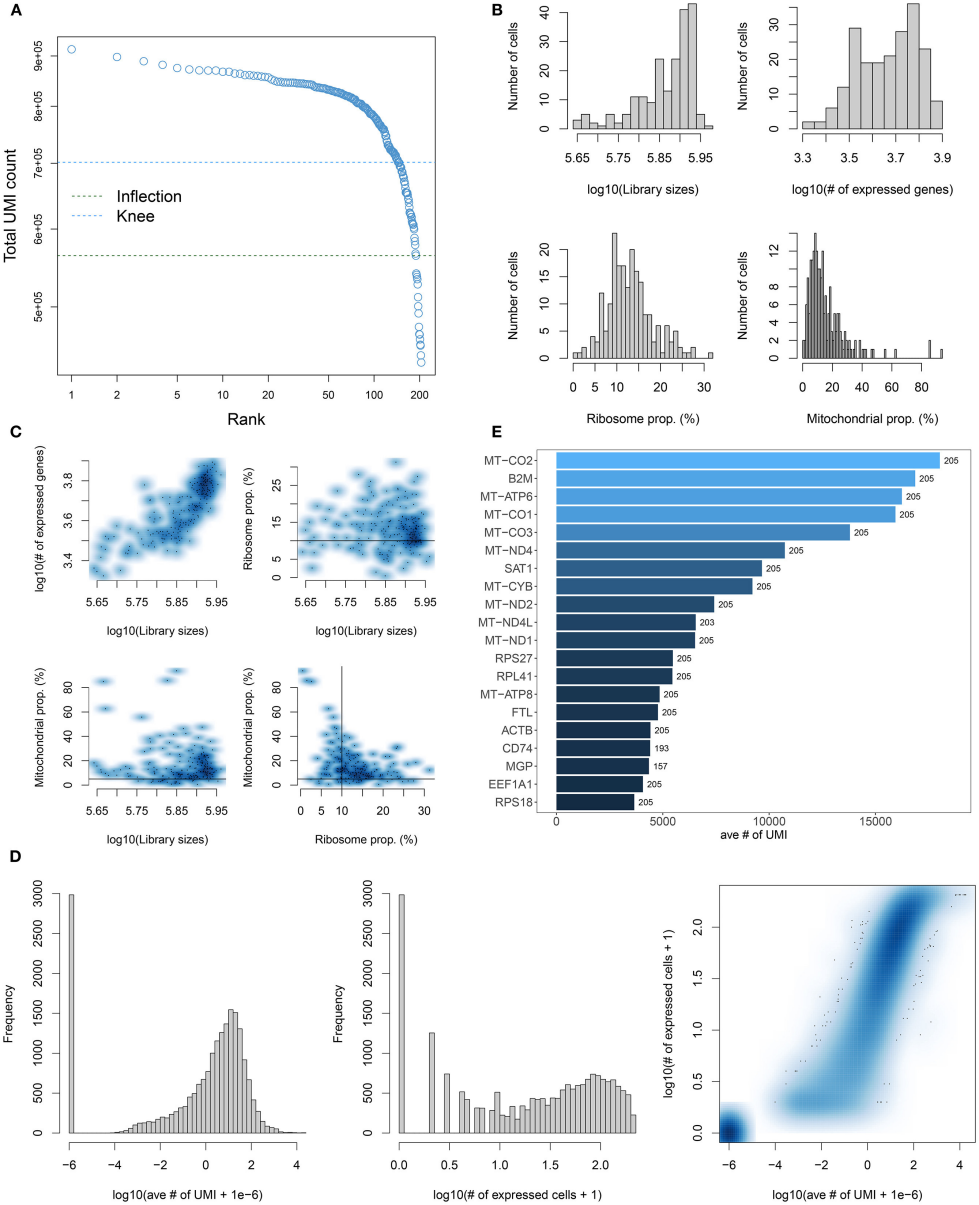
Quality control and preprocessing of single-cell RNA-seq of TNBC cells in the GSE75688 dataset. **(A)** Cell ranking according to the total UMI count. **(B)** The proportion of mitochondrial genes and ribosomal genes in each cell using the calculateQCMetrics of the scater package. **(C)** The ratio of mitochondrial and ribosomal genes expressed in each cell after filtering out cells with expressed genes < 100. **(D)** After filtrating cells with the proportion of mitochondrial genes > 5% and ribosomal genes < 10%, the number of cells was counted. **(E)** The top 20 genes with the high number of expressed cells.

Single-cell RNA-seq data of 205 TNBC cells in the GSE75688 dataset were quality controlled in this study. We calculated each barcode corresponding to each cell using the barcodeRanks function in the DropletUtils package. All cells were ranked by total UMI count (Figure 1A). With the emptyDrops function, barcodes without any gene expression were filtered out, which might not contain any cells. To further filter our cells with low quality, genes expressed in each cell were counted using the calculatQCMetrics in the scater package (Figure 1B). Herein, we filtered out cells with the threshold of the proportion of mitochondrial genes ≤ 5% and ribosomal genes ≥10%. Also, cells expressing <100 genes were filtered out. Then the proportion of mitochondrial and ribosomal genes expressed in each cell was counted again (Figure 1C). Cells with mitochondrial gene expression >5% and ribosomal gene expression <10% were filtered out. After that, the number of cells was counted based on genes again (Figure 1D). No cells were finally removed in this study. The top 20 genes with high number of expressed cells such as MT-CO2, B2M, MT-CO1, and MT-CO3 were displayed in Figure 1E. Intriguingly, genes that encode mitochondrially encoded cytochrome c oxidase were obviously expressed in almost all cells. These data indicated that apoptotic cells could express mitochondrial genes and export these transcripts to the cytoplasm, thereby increasing the proportion of mitochondrial transcripts detected.

### PCA-Based Classification

**Figure 2:**
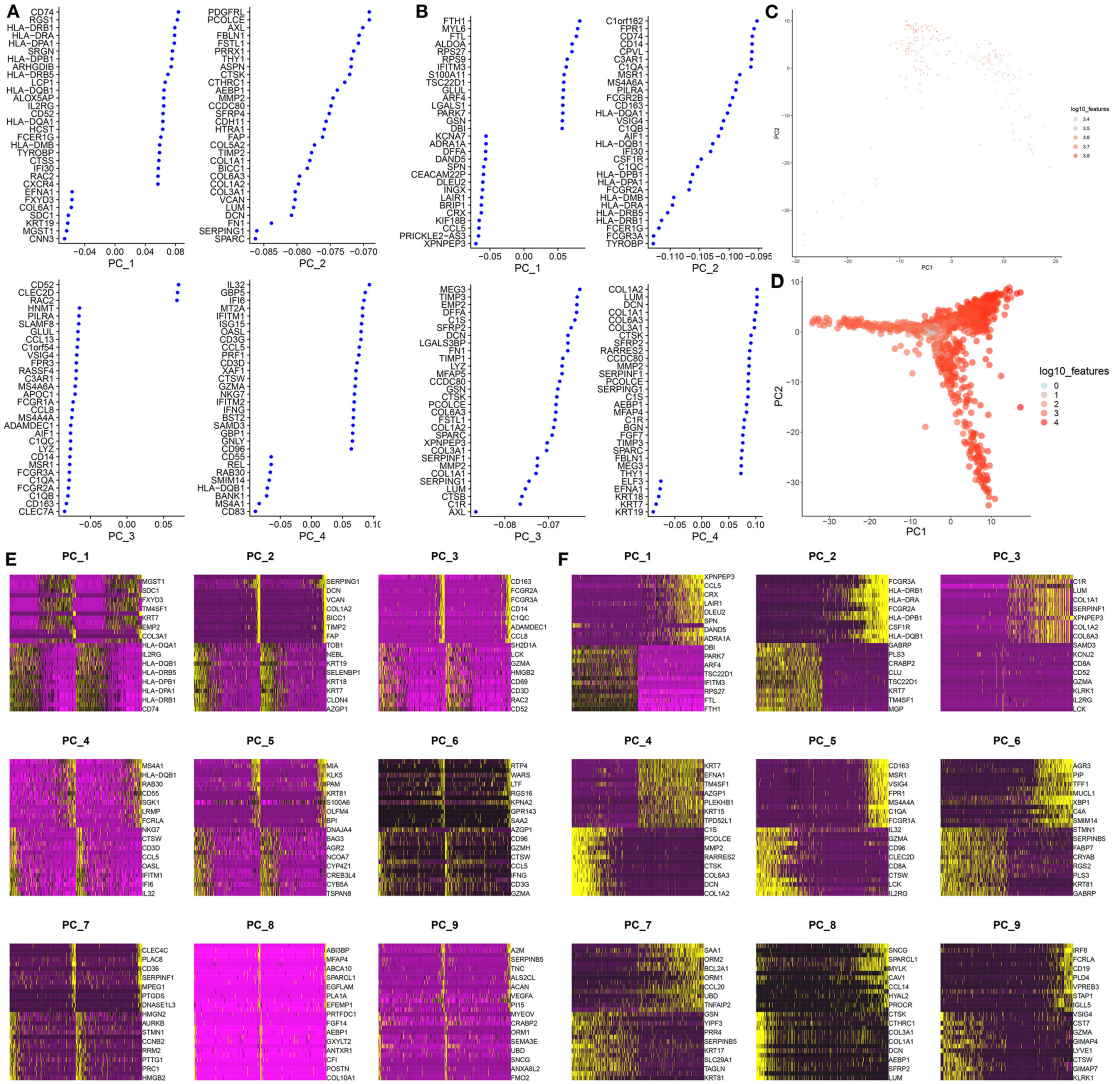
PCA-based classification for single-cell RNA-seq of TNBC cells in the GSE75688 and GSE118389 datasets. **(A, B)** The first four principal components of the normalized data in the GSE75688 and GSE118389 datasets. **(C, D)** The PCA results of all TNBC cells and normal cells in the two datasets. Each dot represents a cell. **(E, F)** Heat maps showing the expression patterns of the top 10 marker genes among different cells in the first nine principal components from the GSE75688 and GSE118389 datasets.

PCA, one of the most extensive dimensionality reduction techniques, can quantify and visualize variability in large data sets. Based on normalized data, the first four PCs were visualized in the GSE75688 (Figure 2A) and GSE118389 (Figure 2B) datasets. As shown in Figures 2C,D, PCA analysis of all genes was separately performed in the two datasets. Since PCA is a linear dimensionality reduction model, the differences between cells were not very significant. Furthermore, we separately showed the top 10 marker genes in the first nine PCs in the GSE75688 (Figure 2E) and GSE118389 (Figure 2F) datasets.

### Cell Clustering Analysis

**Figure 3:**
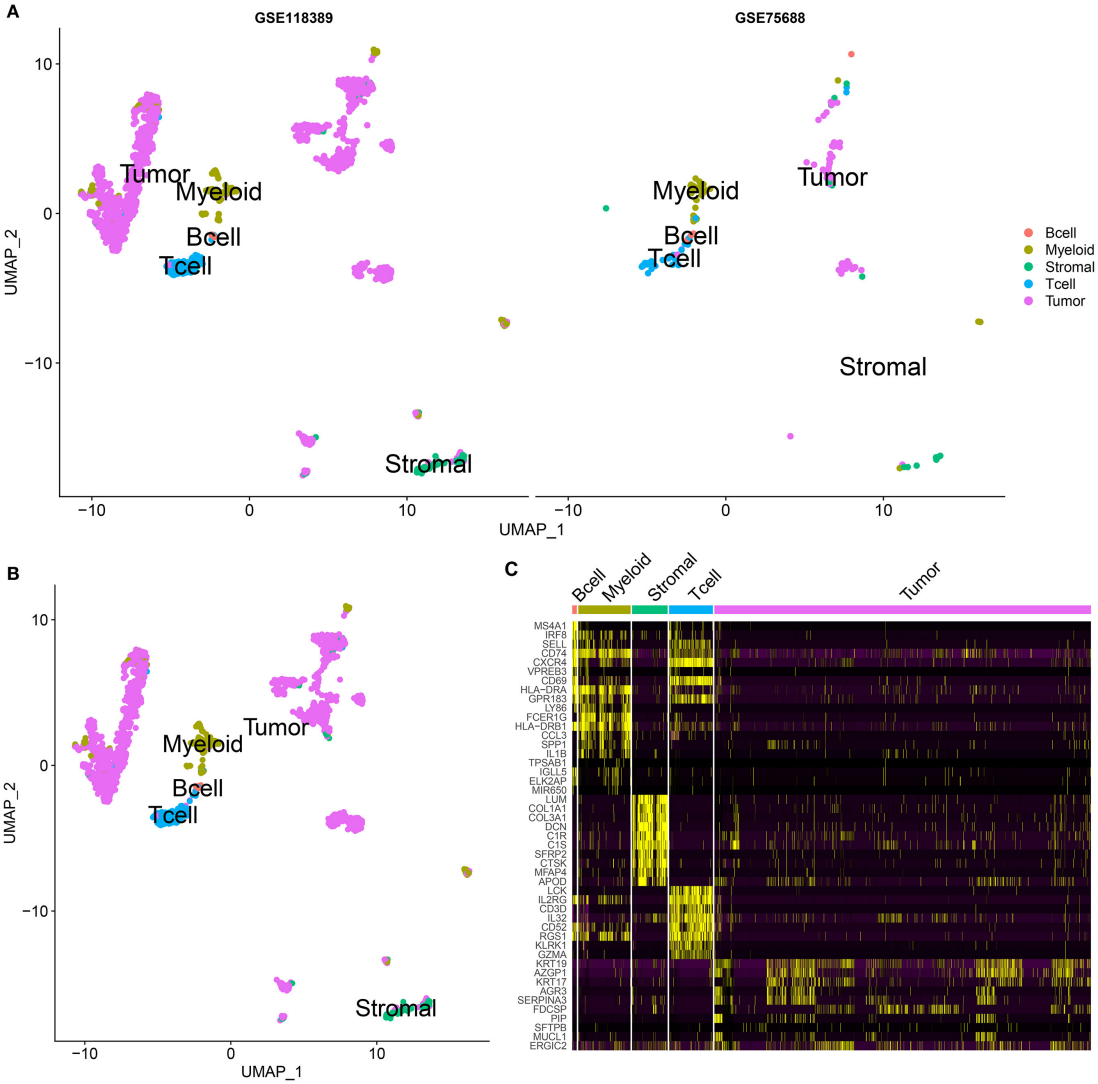
Cell clustering analysis for single-cell RNA-seq of TNBC cells in the GSE75688 and GSE118389 datasets. **(A)** UMAP visualizing the cluster of immune cells (B cells, myeloid and T cells), stromal cells and tumor cells from the GSE75688 and GSE118389 datasets, respectively. Different colors represent different cell clusters. Red: B cells; light green: myeloid cells; dark green: stromal cells; blue: T cells; purple: tumor cells. **(B)** Cell clustering results after integrating the GSE75688 and GSE118389 datasets. **(C)** Heat map showing the expression patterns of the top 10 marker genes in each cell cluster of B cell, myeloid and T cell, stromal cell, and tumor cell after integrating the two datasets.

By UMAP, five cell clusters were visualized based on UMI data, including B cells, myeloid cells, stromal cells, T cells, and tumor cells in the GSE75688 and GSE118389 datasets, respectively (Figure 3A). Then, we integrated the two datasets and Figure 3B displayed the five cell clusters. Supplementary Tables 1–5 listed the marker genes for each cell cluster. Heat map depicted the top 10 marker genes for each cell cluster in the integrated datasets (Figure 3C).

### Cell Cycle of Each Cell Cluster

**Figure 4:**
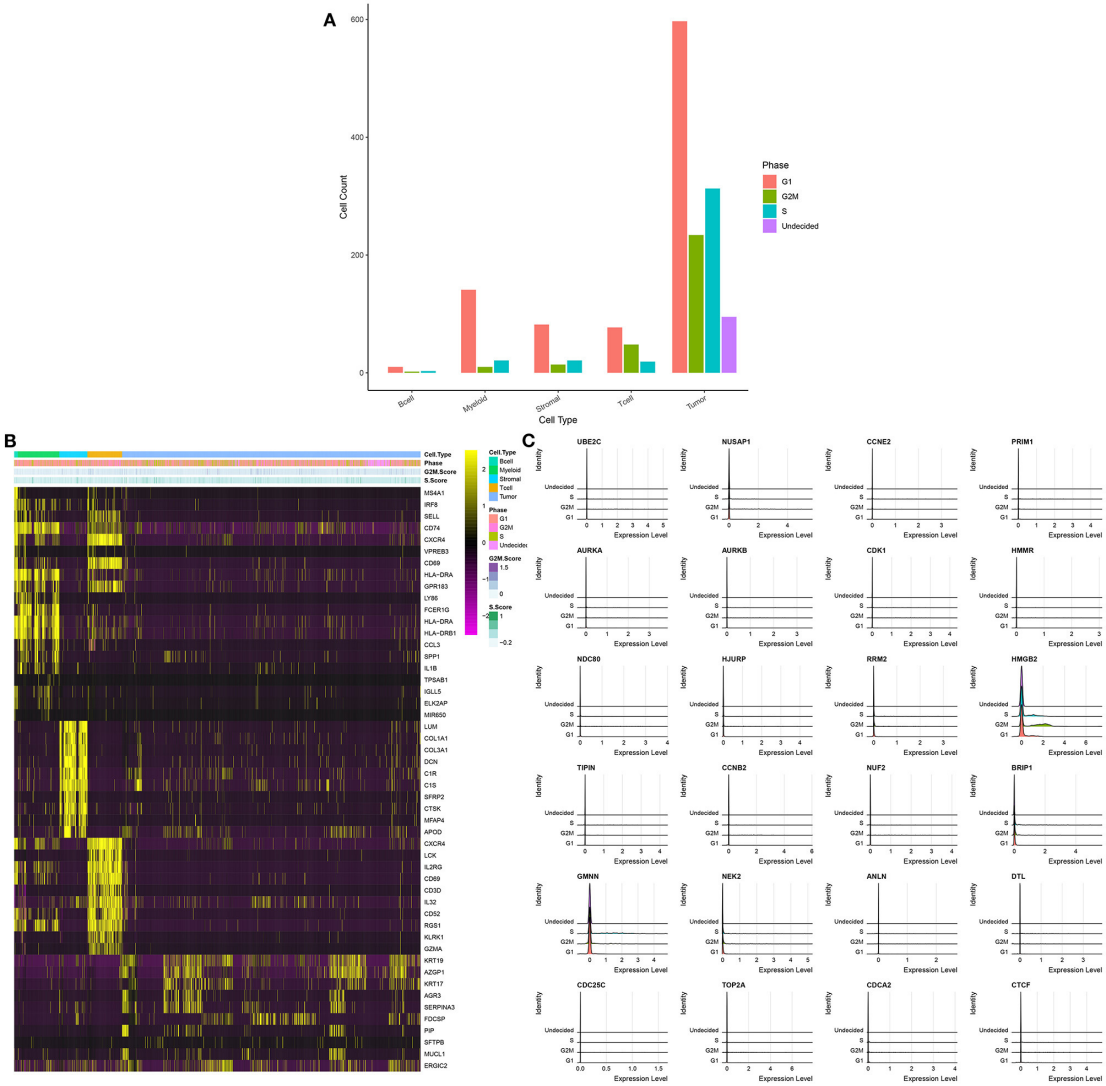
Cell cycle of each cell cluster for single-cell RNA-seq of TNBC cells in the integrated GSE75688 and GSE118389 datasets. **(A)** The distribution of cell cycle phases (G1, G2M, and S) in each cell cluster. **(B)** The heat map depicting the top 10 marker genes of each cell cycle and cell cycle score for each cell cluster. **(C)** Ridge plot visualizing the expression levels of marker genes in different cell cycle phases.

To further analyze the cell cycle status, G1, S, and G2/M phase markers were used to score cell cycle of each cell using the Seurat package in the integrated GSE75688 and GSE118389 datasets. As shown in Figure 4A, we visualized the cell cycle status in each cell cluster. In all cell clusters, the number of cells in G1 phase was the largest. Thus, B cells, myeloid cells, stromal cells, and tumor cells were primarily in G1 phase indicating the high invasiveness of TNBC. Figure 4B displayed the top 10 cell cycle-related markers for each cell cluster. Furthermore, the expression levels of cell cycle-related marker genes in S, G2M, and G1 were visualized in Figure 4C.

### Functional Enrichment Analysis of Genes in Each Cell

**Figure 5:**
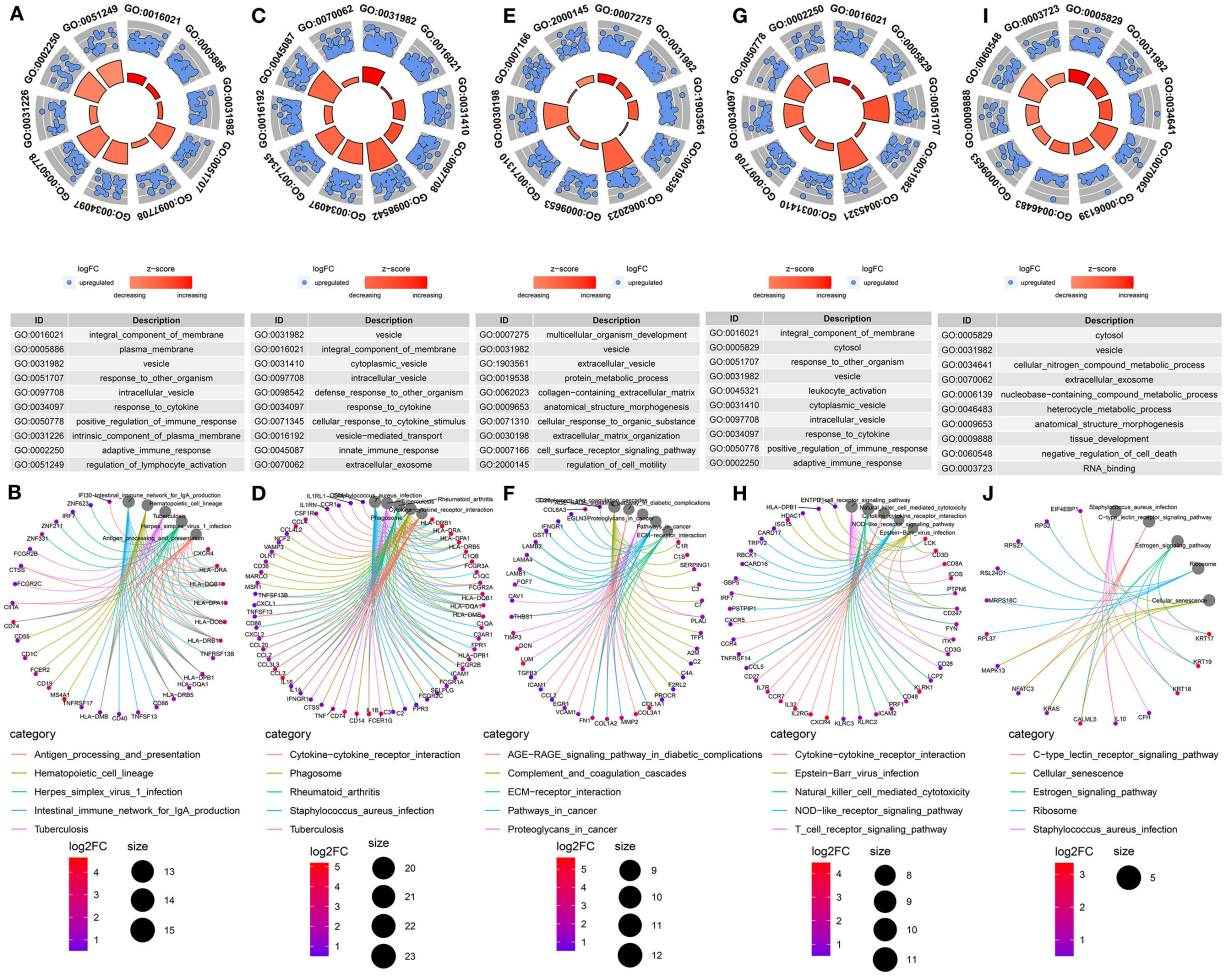
Functional enrichment analysis of marker genes from the integrated GSE75688 and GSE118389 datasets. **(A, B)** GO and KEGG enrichment analysis of marker genes in B cells. **(C, D)** GO and KEGG enrichment analysis of marker genes in myeloid cells. **(E, F)** GO and KEGG enrichment analysis of marker genes in stromal cells. **(G, H)** GO and KEGG enrichment analysis of marker genes in T cells. **(I, J)** GO and KEGG enrichment analysis of marker genes in tumor cells.

GO and KEGG functional enrichment analyses of genes in B cells, myeloid cells, stromal cells, T cells and tumor cells were performed. As expected, the results showed that genes in B cells were significantly associated with plasma membrane and immune response (Figures 5A,B). The genes in myeloid cells were mainly enriched in immune system response (Figures 5C,D). For stromal cells, the genes were in association with organism development and extracellular matrix (Figures 5E,F). As shown in Figures 5G,H, the genes in T cells were significantly related with T cell receptor signaling pathway. GO functional enrichment analysis results showed that the genes in tumor cells were mainly enriched in tissue or cell development (Figure 5I). Furthermore, according to KEGG enrichment analysis results, these genes were significantly associated with estrogen signaling pathway, which could be involved in the development of TNBC (Figure 5J).

### Ligand–Receptor Signaling Network

**Figure 6:**
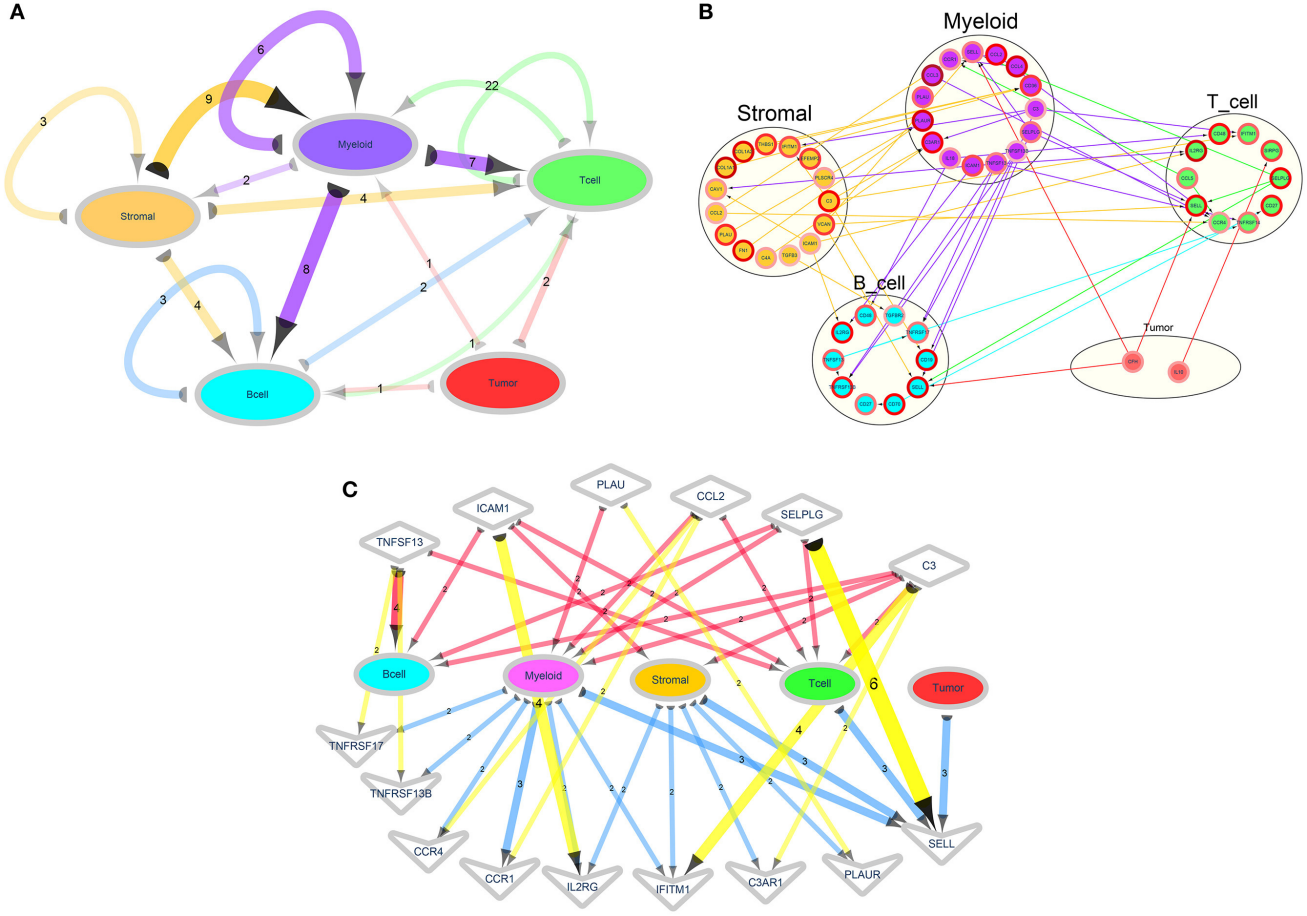
Ligand–receptor signaling networks. **(A)** A cell type-based ligand receptor network. The semicircle represents ligand and the arrow points to the receptor. The number on the side indicates the number of ligand–receptor relationship pairs. The color of the edge and the corresponding cell type refer to the cell type to which the ligand gene belongs. The depth of the color represents the number of ligand–receptor relationships. **(B)** A detailed network of the ligand receptors in each cell type. The arrows from the ligands point to the receptors. The color of the edge corresponds to the cell type to which the corresponding ligand type refers to the ligand gene. The color of the edges of the nodes indicates that the log2FC of the marker genes. **(C)** A network diagram showing the relationship between the designated ligand receptor and each cell. The semicircle represents the ligand, and the arrow points to the receptor. The numbers on the edges indicate the number of ligand–receptor relationships, and the shades of the edges represents the number of ligand–receptor relationships. Diamond nodes represent ligands and V nodes represent receptors.

Intercellular communication networks were conducted based on ligand–receptor pairs. Herein, ligand was known as sender and receptor as receiver. Using the above five cell lineages including B cells, T cells, tumor cells, stromal cells, and myeloid cells, we calculated the number of major signal pairs that could communicate within and across lineages (Figure 6A). Detailed network diagram of ligand receptors in each cell type is shown in Figure 6B. After excluding relationships with the count = 1 and the genes that were not directly related to the cell and had no ligand–receptor relationships a sub-network was constructed, as shown in Figure 6C.

### Correlation Between Tumor-Infiltrating Immune Cells and TNBC Patients' Prognosis

**Figure 7:**
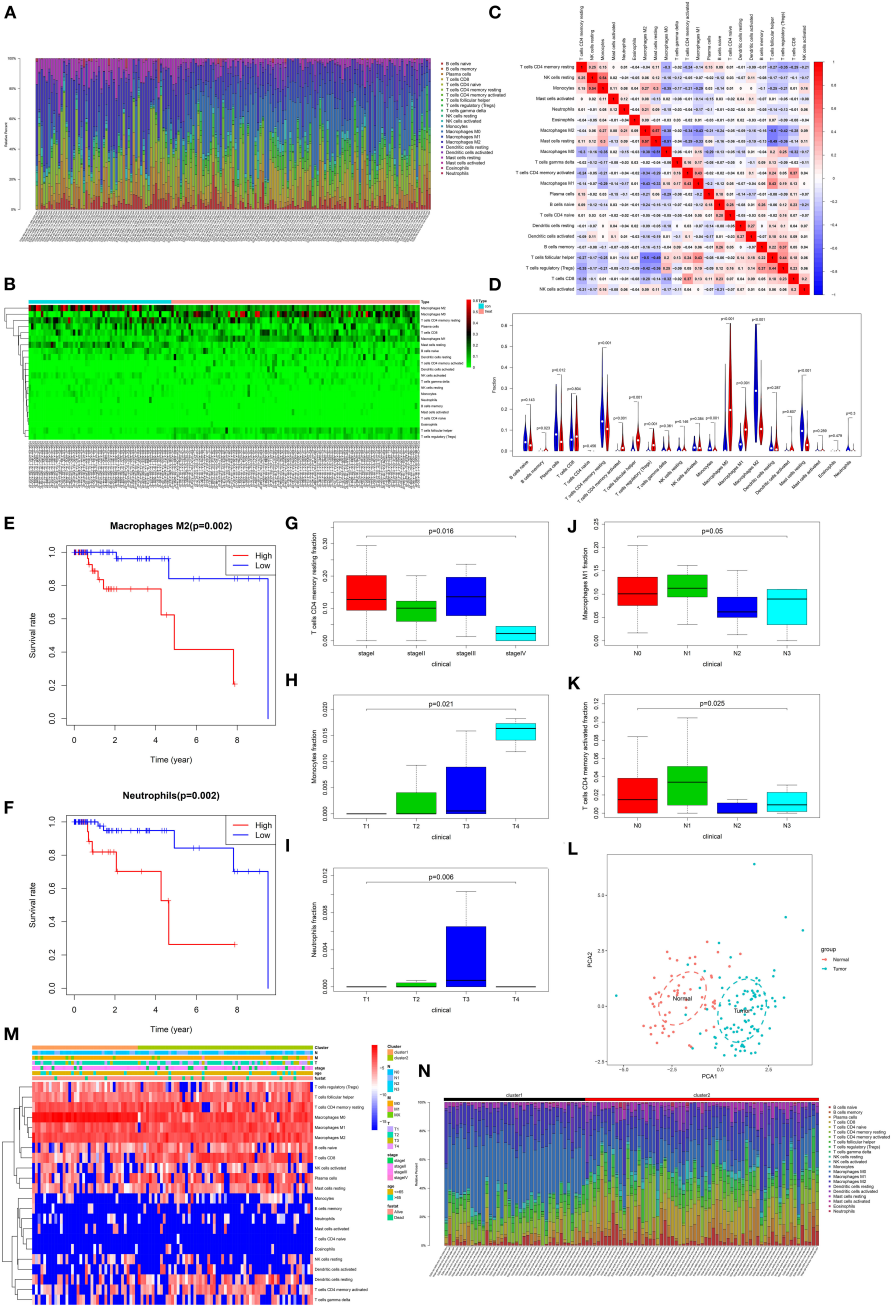
Correlation between tumor-infiltrating immune cells and TNBC patients' prognosis. **(A)** Histogram showing the fractions of 22 tumor-infiltrating immune cells in 58 normal adjacent tissues and 101 TNBC tissues. Different colors in the column represent different immune cells. The height of the column indicates the fractions of each immune cell. The higher the height, the higher the proportion of the immune cells in the tissue. **(B)** Heatmap showing the fractions of different immune cells in TNBC tissues and adjacent normal tissues. Green indicates a low percentage and red indicates a high percentage. **(C)** Heatmap depicting the correlation between different immune cells. Red represents positive correlation and blue represents negative correlation. The darker the color, the stronger the correlation. **(D)** Violin plots showing the difference in fractions of these immune cells between TNBC tissues and adjacent normal tissues. Red suggests TNBC tissues and blue suggests adjacent normal tissues. Overall survival for TNBC patients between high and low fractions of M2 macrophages **(E)** and neutrophils **(F)**. The differences in fractions of T cells CD4 memory resting **(G)**, monocytes **(H)**, neutrophils **(I)**, macrophages M1 **(J)** and T cells CD4 memory activated **(K)** in all TNBC patients. **(L)** The PCA results of immune cells. Red dot represents normal samples and blue represents TNBC samples. **(M)** Heatmap depicting the fractions of immune cells in the two clusters, TNM, stage, age, and Fustat. **(N)** Histogram showing the fractions of 22 tumor-infiltrating immune cells in cluster1 and cluster2.

Through estimation of the abundance cell types in mixed cell populations based on TNBC RNA-seq data using CIBERSORT, the proportion of 22 immune cells in each sample was calculated. With the threshold of *p* < 0.05, immune cells from 58 normal adjacent tissues and 101 TNBC tissues were obtained. In Figure 7A, the relative percent of different immune cells was shown. We found that the distribution of various types of immune cells was different between different TNBC tissues, indicating the heterogeneity among different tissues. The heatmap depicted the difference in expression patterns of different immune cells between adjacent normal tissues and TNBC tissues (Figure 7B). As shown in correlation analysis results, positive correlation between NK cell resting and monocytes (*r* = 0.54), positive correlation between M2 macrophage and mast cell resting (*r* = 0.57), negative correlation between M0 macrophage and mast cell resting (*r* = −0.51) and negative correlation between T cells follicular helper and M2 macrophage (*r* = −0.50) were found (Figure 7C). In Figure 7D, we found that the fractions of B cell memory (p = 0.023), T cells CD4 memory activated (*p* < 0.001), T cells follicular helper (*p* < 0.001), T cells regulatory (Tregs; *p* < 0.001), macrophages M0 (*p* < 0.001), macrophages M1 (*p* < 0.001) were significantly higher in TNBC tissues than those in adjacent normal tissues. Furthermore, the fractions of plasma cells (*p* = 0.012), T cells CD4 memory resting (*p* < 0.001), monocytes (*p* < 0.001), macrophages M2 (*p* < 0.001) and mast cells resting (*p* < 0.001) were significantly lower in TNBC tissues compared to those in adjacent normal tissues.

To explore the prognostic value of these immune cells for TNBC patients, we performed overall survival analysis. Among 22 immune cells, the high fractions of M2 macrophages (*p* = 0.002) and neutrophils (*p* = 0.002) were significantly associated with shorten survival time than their low fractions (Figures 7E,F). Moreover, we found that the fractions of T cells CD4 memory resting were significantly associated with stages (*p* = 0.016; Figure 7G). The fractions of monocytes significantly increased as pathological T stage (*p* = 0.021; Figure 7H). Compared to other pathological T stages, neutrophils had the highest fractions in pathological T3 stage (*p* = 0.006; Figure 7I). Furthermore, the fractions of M1 macrophages (*p* = 0.05; Figure 7J) and T cells CD4 memory activated (*p* = 0.025; Figure 7K) were significantly associated with pathological N stage.

Our PCA results showed that these immune cells could significantly distinguish TNBC tissues into adjacent normal tissues (Figure 7L). The ConsensusClusterPlus in R package was used for tumor stages based on immune cells. Based on the elbow and gap statistics methods, the optimal number of clusters was determined. For a balanced partition, *k* was set as 2. As shown in Figure 7M, the samples were sorted according to cluster, T, N, M, stage, age, and survival status, furthermore, the fractions of immune cells among different groups were displayed. Moreover, there were significant differences in the fractions of different immune cells between cluster1 and cluster2 (Figure 7N).

### Validation of Marker Genes in TNBC From TCGA Database

**Figure 8:**
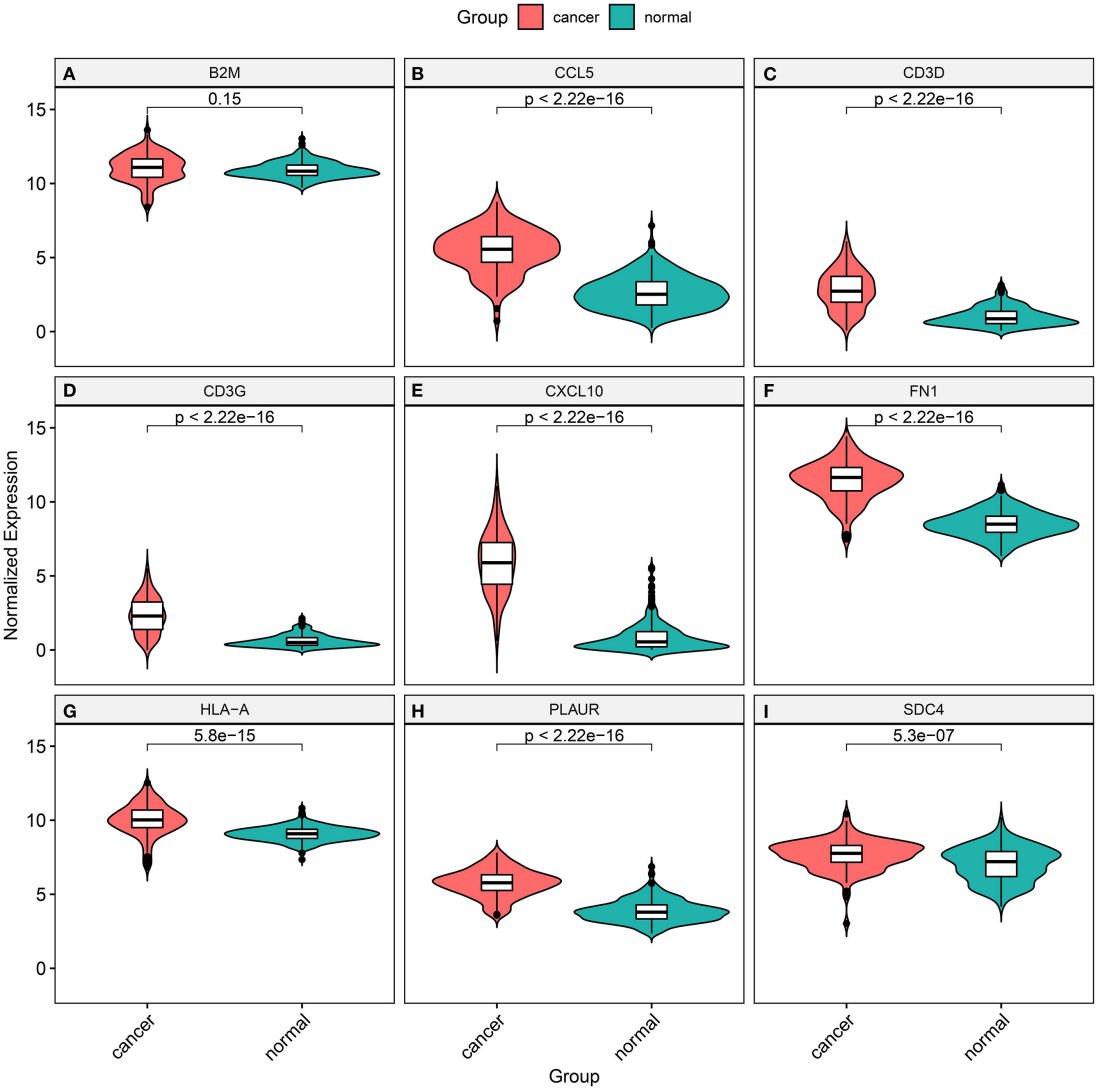
Validation of the expression of marker genes between TNBC and normal tissues from TCGA database. **(A)** B2M (*p* = 0.15); **(B)** TCCL5 (*p* < 2.22e–16); **(C)** CD3D (*p* < 2.22e– 16); **(D)** CD3G (*p* < 2.22e–16); **(E)** CXCL10 (*p* < 2.22e–16); **(F)** FN1 (*p* < 2.22e–16); **(G)** HLA-A (*p* = 5.8e–15); **(H)** PLAUR (*p* < 2.22e–16); and **(I)** SDC4 (*p* = 5.3e–07).

The expression of marker genes was validated between TNBC and normal tissues from TCGA database. In Figure 8A, there was no statistical significance in B2M expression between TNBC and normal tissues (*p* = 0.15). The expression of TCCL5 (*p* < 2.22e–16; Figure 8B), CD3D (*p* < 2.22e–16; Figure 8C), CD3G (*p* < 2.22e–16; Figure 8D), CXCL10 (*p* < 2.22e–16; Figure 8E), FN1 (*p* < 2.22e–16; Figure 8F), HLA-A (*p* = 5.8e–15; Figure 8G), PLAUR (*p* < 2.22e–16; Figure 8H) and SDC4 (*p* = 5.3e–07; Figure 8I) were distinctly highly expressed in TNBC tissues in comparison to normal tissues.

## Discussion

Most TNBCs possess shared histological and molecular characteristics. Nevertheless, TNBC is characterized by inter-tumor and intra-tumor heterogeneity (15). Multiple evidences suggest that the intratumoral diversity of TNBC is not only involved in the pathogenesis, but also affects chemotherapy resistance, metastasis, and poor clinical outcomes (4, 16, 17). Due to the heterogeneity of TNBC at the molecular level, identification of effective prognostic markers and therapeutic target is challenging. In this study, to explore the intercellular heterogeneity in TNBC, we analyzed single-cell RNA-seq data of TNBC cells by integrating the GSE75688 and GSE118389 datasets.

Our single-cell RNA-seq analysis results showed that TNBC was composed of different proportions of tumor cells, stromal cells, and immune cells (B cells and T cells), which was consistent with previous studies (18). Single-cell RNA-seq provides support for characterizing the different functional states of a single cell. It has been accepted that scRNA-seq could be used to detect and quantify transcriptional changes at a single cell level (19). Cell cycle is a main driving force of transcriptional heterogeneity and cell-fate decisions. G1 phase is a sensitive period of cell fate. In addition, the cell cycle is known to be involved in various biological processes, such as cell differentiation (20) and tumorigenesis (21, 22). Therefore, we accurately identified the cell cycle phases of each cell. Different cell clusters exhibit different cell cycles, revealing that TNBC was indeed heterogeneous, and the different cell clusters participated disproportionately in the tumor.

It has been confirmed that cell differentiation and cell-fate decision may be controlled via communicating with neighboring cells (14). In cancers including TNBC, intercellular communication mediates the activities of different cell types are involved in complex biological processes, such as invasion, cell cycle, and immune response (18, 23, 24). It has been widely accepted that most cells can express a variety of ligands and receptors to conduct a highly connected ligand–receptor signaling pathway network. In this study, we constructed ligand–receptor signaling networks for TNBC based on marker genes of five types of cells. In this study, we compiled ligand–receptor relationship pairs based on literature support and constructed an intercellular communication network, which revealed extensive and interlineage signals.

It is recognized that changes in the composition of cells are the basis for the various physiological states of complex tumor tissues. Especially in malignant tumors, the level of invasive immune cells is closely related to tumor progression and clinical outcome (8). Thus, in this study, we used CIBERSORT to accurately assess the relative scores of different immune cell types in TNBC tissues. Compared to current methods for studying cell heterogeneity (such as immunohistochemistry and flow cytometry), CIBERSORT is free of limited phenotypic markers or cell loss or damage due to tissue breakdown. The heterogeneity in the fractions of 22 immune cells was found in TNBC tissues. As expected, there was a significant difference in the fractions of these immune cells between adjacent normal tissues and TNBC tissues. The intratumoral heterogeneity may affect prognosis of TNBC. Therefore, we analyzed the prognostic value of these immune cells. We found that M2 macrophages and neutrophils were significantly associated with clinical outcomes of TNBC. As previous studies, M2 macrophages could regulate PD-1/PD-L1 expression in the tumor microenvironment, thereby affecting the effect of targeted treatment (25). A retrospective study found that an increase in the ratio of neutrophils to lymphocytes was associated with a poor prognosis in TNBC patients undergoing chemotherapy (26). Furthermore, the fractions of T cells (27), monocytes (28), neutrophils (29), and M1 macrophages (30) were significantly correlated with clinical characteristics of TNBC. More importantly, our PCA results showed that these immune cells could significantly distinguish TNBC tissues into adjacent normal tissues. These findings revealed the prognostic value of the tumor immune microenvironment for TNBC.

In this study, we systematically characterized the tumor heterogeneity of TNBC based on single-cell RNA-seq and transcriptome RNA-seq. Despite the limitations of retrospective studies, our study gained a deeper understanding of the complex interactions between tumor cells and their microenvironment (especially immune cells).

## Conclusion

In this study, we unraveled the heterogeneity of tumor cells and their microenvironment. Furthermore, clinical implications of tumor microenvironment components were characterized for TNBC. Our study may provide an evidence for TNBC immunotherapy.

## Data Availability Statement

The datasets presented in this study can be found in online repositories. The names of the repository/repositories and accession number(s) can be found in the article/Supplementary Material.

## Author Contributions

JS conceived and designed the study. KJ conducted most of the experiments and data analysis, and wrote the manuscript. MD and CL participated in collecting data and helped to draft the manuscript. All authors reviewed and approved the manuscript.

## Conflict of Interest

The authors declare that the research was conducted in the absence of any commercial or financial relationships that could be construed as a potential conflict of interest.

## Supplementary Material

Supplementary Table 1 Marker genes in B cells.

Supplementary Table 2 Marker genes in myeloid cells.

Supplementary Table 3 Marker genes in stromal cells.

Supplementary Table 4 Marker genes in T cells.

Supplementary Table 5 Marker genes in tumor cells.

## Abbreviations

TNBC: triple negative breast cancer
scRNA-seq: single-cell RNA sequencing
ER: estrogen receptor
PR: progesterone receptor
UMI: unique molecular identifiers
PCA: principal component analysis
PCs: principal components
UMAP: Uniform Manifold Approximation and Projection
tSNE: t-distributed stochastic neighbor embedding
FC: fold change
GO: Gene Ontology
BRCA: breast invasive carcinoma
TCGA: The Cancer Genome Atlas
BP: biological process
CC: cellular component
MF: molecular function.
